# Molecular analysis of zoonotic pathogens in free-ranging six-banded armadillos (*Euphractus sexcinctus*) from the Brazilian semiarid region

**DOI:** 10.1590/S1984-29612025002

**Published:** 2025-01-13

**Authors:** Ilanna Vanessa Pristo de Medeiros Oliveira, José Artur Brilhante Bezerra, Gabriela Hémylin Ferreira Moura, Ana Carolina Yamakawa, Mariana Guimarães Nilsson, Jéssica da Silva Ferreira, Amanda Haisi, Felipe Fornazari, Hélio Langoni, João Marcelo Azevedo de Paula Antunes

**Affiliations:** 1 Hospital Veterinário Jerônimo Dix-Huit Rosado Maia, Universidade Federal Rural do Semi-Árido – UFERSA, Mossoró, RN, Brasil; 2 Centro de Pesquisa em Zoonoses – NUPEZO, Departamento de Higiene Veterinária e Saúde Pública, Faculdade de Medicina Veterinária e Zootecnia, Universidade Estadual Paulista – UNESP, Botucatu, SP, Brasil; 3 Laboratório Multiusuário de Biologia Molecular, Departamento de Parasitologia Animal, Instituto de Medicina Veterinária, Universidade Federal Rural do Rio de Janeiro – UFRRJ, Seropédica, RJ, Brasil; 4 Instituto de Biotecnologia, Universidade Estadual Paulista – UNESP, Botucatu, SP, Brasil

**Keywords:** Zoonoses, Chagas disease, leishmaniasis, leptospirosis, toxoplasmosis, Zoonoses, Doença de Chagas, leishmaniose, leptospirose, toxoplasmose

## Abstract

This study investigated infection by *Leishmania* spp., *Leptospira* spp., *Toxoplasma gondii*, and *Trypanosoma cruzi* in six-banded armadillos (*Euphractus sexcinctus*) from the semiarid region of northeastern Brazil. Twenty specimens of *E. sexcinctus* were captured alive by wildlife veterinarians from their natural habitats in different locations. The animals were euthanized following induction of anesthesia, and different biological samples were collected. Infection with four pathogens was subsequently evaluated: *Leishmania* infection was investigated by spleen and liver Polymerase Chain Reaction (PCR); *Leptospira* spp. infection was evaluated by kidney PCR and serologically by microscopic agglutination test; *T. gondii* infection was assessed by PCR of the heart, lung, and spleen; and *T. cruzi* infection was investigated by heart and whole blood PCR and hemoculture. All tests presented negative results apart from whole blood PCR to detect *T. cruzi*, which was positive in one of the 20 animals tested and confirmed by genetic sequencing. It is important to highlight that this is the first study comprising a molecular investigation of different zoonotic pathogens in six-banded armadillos, and the findings reported here bring new and important knowledge regarding zoonotic diseases in this species.

## Resumo

Este estudo objetivou investigar a infecção por *Leishmania* spp., *Leptospira* spp., *Toxoplasma gondii* e *Trypanosoma cruzi* em tatus-peba (*Euphractus sexcinctus*) de vida livre provenientes da região semiárida do nordeste brasileiro. Vinte espécimes de *E. sexcinctus* foram capturados vivos de seus habitats naturais por veterinários de animais silvestres em diferentes locais. Após anestesia, os animais foram eutanasiados e diferentes amostras biológicas foram coletadas. Os quatro patógenos foram avaliados da seguinte forma: a infecção por *Leishmania* foi investigada por meio da Reação em Cadeia da Polimerase (PCR) de baço e fígado; a infecção por *Leptospira* spp. foi avaliada por meio da PCR de rim e sorologicamente através do teste de aglutinação microscópica; a infecção por *T. gondii* foi feita por meio de PCR de coração, pulmão e baço; e a infecção por *T. cruzi* foi investigada por meio da PCR de coração e sangue total e por hemocultura. Todos os testes realizados tiveram resultados negativos, exceto a PCR de sangue total para detectar *T. cruzi*, a qual foi positiva em apenas um animal e confirmado por meio de sequenciamento genético. É importante enfatizar que esse é o primeiro estudo com investigação molecular de diferentes patógenos zoonóticos em tatus-peba, e os achados aqui reportados trazem novo e importante conhecimento a respeito de doenças zoonóticas nessa espécie.

The six-banded armadillo (*Euphractus sexcinctus*) is a wild mammal species used as a protein source for human consumption. Illegal hunting and captive breeding of this species are common in local communities in Northeastern Brazil (Barboza et al., 2016). However, recent studies have linked these animals to a variety of zoonotic diseases, including an alarmingly high rate of *Mycobacterium leprae* infection (Ferreira et al., 2020), and serological evidence of exposure to *Toxoplasma gondii*, *Leishmania* spp., and *Leptospira* spp. (Barbosa et al., 2020) in free-ranging armadillos from Northeastern Brazil. The infection of *E. sexcinctus* by *Trypanosoma cruzi*, the etiological agent of Chagas disease, was also reported in the same region (Martins et al., 2015). The close contact of humans with armadillos during hunting, breeding, and meat consumption could represent a risk of transmission of these pathogens (Deps et al., 2020). As such, this study aimed to investigate the rates of infection by *T. gondii*, *Leishmania* spp., *Leptospira* spp., and *T. cruzi* in armadillos to clarify the role of these animals in the zoonotic disease epidemiological chain.

**Figure 1:**
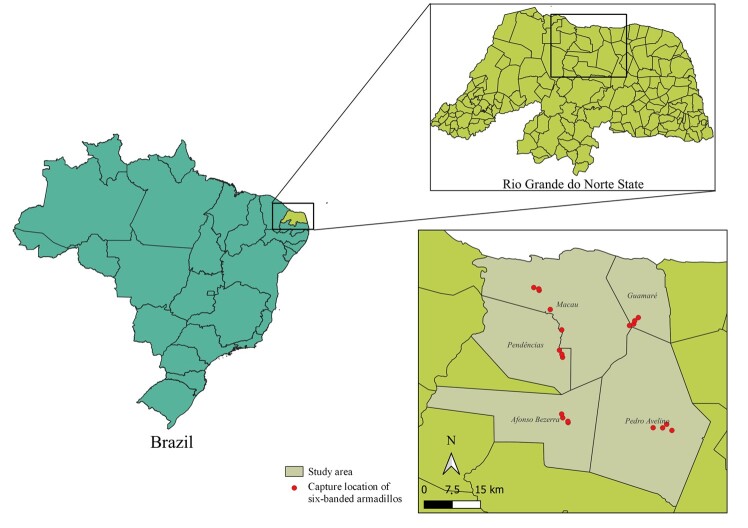
Map showing the detailed locations where the six-banded armadillos (*Euphactus sexcintus*, *n* = 20) utilized in this study were captured.

This study was conducted in Rio Grande do Norte State, Brazil, from May to June 2016. Twenty specimens of *E. sexcinctus* were captured alive from their natural habitats by wildlife veterinarians from twenty distinct locations within five rural municipalities (Pendências, Afonso Bezerra, Macau, Pedro Avelino e Guamaré) (Figure 1).

All animals were anesthetized by simultaneous administration of tiletamine hydrochloride and zolazepam hydrochloride (Zoletil^®^ 50, Virbac; 4 mg/kg intramuscularly) (Ferreira et al., 2020). Blood samples were collected by cardiac puncture using a 22 G 1 1/4-gauge needle attached to a 10 mL syringe. Part of the collected blood sample of each animal was transferred to a 13 × 75 mm volume 3.0 mL BD Vacutainer^®^ tube containing 3.4 mg spray-coated K_2_EDTA (Becton Dickinson, New Jersey, USA) to be used for hemoculture of *T. cruzi* and molecular assays. The remaining volume was transferred to a 16 × 100 mm, 10 mL volume, plain/no additive, BD Vacutainer^®^ tube (Becton Dickinson, New Jersey, USA) and allowed to clot for 1 hour at room temperature (25 °C), followed by centrifugation (1500 x g for 15 min at 4 °C) to collect fully separated serum for serological testing of *Leptospira* spp. After the confirmation of loss of corneal reflex in the previously anesthetized animals, euthanasia was performed by the administration of potassium chloride via the femoral vein at a dose of 2.56 mEq/Kg (1 mL/kg of a 19.1% solution). Necropsy was subsequently performed, and samples of the lungs, spleen, liver, and kidneys were collected for molecular tests. Whole blood, tissue, and serum samples were stored at -80 °C in Eppendorf tubes until serological and molecular analysis.

From the 20 armadillos included in this research, we collected following types and numbers of samples: whole blood (*n* = 20), serum (*n* = 20), spleen (*n* = 20), liver (*n* = 20), heart (*n* = 10), lung (*n* = 10) and kidney (*n* = 10). A lower number of heart, lung, and kidney samples were included as samples from 10 animals were assigned to a different study.

All molecular and serological tests were conducted at the Zoonosis Diagnosis Service of the São Paulo State University (UNESP), FMVZ-UNESP, Campus of Botucatu, SP, Brazil. DNA extraction from blood samples was performed using Illustra™ blood GenomicPrep Mini Spin kit (GE Healthcare, Chicago, IL, USA), while DNA extraction from tissue samples was conducted using the Illustra™ tissue and cells GenomicPrep Mini Spin kit (GE Healthcare, Chicago, IL, USA), following the manufacturers’ recommendations. The purified DNA samples were eluted in 100 μL. The quality of the DNA was assessed by the concentration, which was then adjusted up to 100 ng/μL, and by the absorbance ratios of 260/280 and 260/230 nm using a Nanovue spectrophotometer (GE Healthcare Life Sciences, Chicago, IL, USA). The presence of amplifiable DNA was confirmed by conventional Polymerase Chain Reaction (PCR) using the endogenous mammalian gene that encodes glyceraldehyde-3-phosphate dehydrogenase (GAPDH) (Birkenheuer et al., 2003).

Conventional PCR assays were performed to identify the presence of the genetic material of *T. gondii*, *T. cruzi*, *Leishmania* spp., and *Leptospira* spp. The primers and samples used in each assay are shown in Table 1. In all PCR reactions, ultrapure DNAse/RNAse-Free distilled water was used as a negative control. Positive controls were also included in each PCR reaction: *Leishmania* spp. PCR – DNA sample extracted from *L. major* (MHOM/SU/1973/5-ASKH strain); *T. cruzi* PCR – DNA sample extracted from of *T. cruzi* (“Y” strain); *Leptospira* spp. PCR – DNA sample extracted from antigen kept at EMJH (serovar Hardjo); *T. gondii* PCR – DNA sample extracted from tachyzoites of the *T. gondii* RH strain. The amplification steps were carried out in a thermal cycler (Mastercycler pro; Eppendorf, Hamburg, Germany) following previously described protocols (Moser et al., 1989; Mérien et al., 1992; Homan et al., 2000; Volpini et al., 2004). Electrophoresis was performed in a 2% agarose gel stained with Nancy-520^®^ (Sigma-Aldrich, St. Louis, MO, USA) in 1x TBE buffer, at 80 Volts (V) for 20 min followed by 100 V for 20 min, with DNA MW Marker 100-bp Ladder^®^ (Sinapse Biotecnologia, Tatuapé, SP, Brazil). The resulting gel was visualized using an ultraviolet transilluminator.

**Table 1:** Primers and samples used in the study to test for the presence of infectious agents by PCR in six-banded armadillos (*Euphractus sexcinctus*) from Rio Grande do Norte State, Brazil.

Target species	Sample type	Number of samples	PCR Primer	Product size (pb)	PCR conditions	Reference
*Leishmania* spp.	Liver	20	150: 5’-GGG(G/T)AGGGGCGTTCT(C/G)CGAA-3’ 152: 5’-(C/G)(C/G)(C/G)(A/T)CTAT(A/T)TTACACCAACCCC-3’	120	95 °C, 5 min; 29 cycles (95 °C 60 sec, 55 °C 30 sec, 72 °C 10 sec); 72 °C, 5 min	Volpini et al. (2004)
	Spleen	20				
*Leptospira* spp.	Kidney	10	Lep1: 5’GGCGGCGCGTCTTAAACATG3’ Lep2: 5’TTCCCCCCATTGAGCAAGATT3’	331	94 °C, 4 min; 30 cycles (94 °C 1 min, 63 °C 1.5 min, 72 °C 2 min); 72 °C, 10 min	Mérien et al. (1992)
*Toxoplasma gondii*	Heart	10	TOX4: 5’CGCTGCAGGGAGGAAGACGAAAGTTG3’ TOX5: 5’CGCTGCAGACACGTGCATCTGGATT3’	529	94 °C, 7 min; 35 cycles (94 °C 1 min, 60 °C 1 min, 72 °C 1 min); 72 °C, 10 min	Homan et al. (2000)
	Lung	10				
	Spleen	20				
*Trypanosoma cruzi*	Heart	10	TCZ1: 5’CGAGCTCTTGCCCACACGGGTGCT3’ TCZ2: 5’CCTCCAAGCAGCGGATAGTTCAGG3’	188	94 °C, 10 min; 25 cycles (94 °C 30 sec, 55 °C 1 min, 72 °C 1.5 min); 72 °C, 3 min	Moser et al. (1989)
	Whole blood	20				

Hemoculture (HC) was additionally conducted to investigate the infection by *T. cruzi* and the microscopic agglutination test (MATLepto) was performed to detect anti-*Leptospira* spp. antibodies. According to the protocol outlined by Alves et al. (2016), HC was conducted under sterile conditions through the inoculation of 0.3 ml of each blood sample in an NNN (Novy-McNeal-Nicolle) medium with a Liver Infusion Tryptose (LIT) overlay. The tubes were examined biweekly for five months. For the MATLepto, a collection of 28 antigens previously described by Fornazari et al. (2018) was used, with 1:100 considered as the cut-off point. Details on the MAT-Lepto protocol were described by Fornazari et al. (2012).

All PCR assays to investigate zoonotic pathogens in the armadillos were negative, except for the PCR to detect *T. cruzi* in blood samples, which was positive in only one out of the 20 animals tested. The results of HC and MATLepto were also negative.

The amplified products were purified using the NZYGelpure Kit (NZYTech, Lisbon, Portugal) according to the manufacturer’s instructions and sequenced with 7 μL of the purified sample added to a solution containing 3 μL of each primer (3.2 pmol). Sequencing took place in the forward and reverse directions with the TCZ1 and TCZ2 primers used in PCR. Sequencing took place using the BigDyeTM Terminator v3.1 Cycle Sequencing Kit (Applied Biosystems, Foster City, CA, USA) by Sanger Method using 3500 Genetic Analyzer (Applied Biosystems, Foster City, CA, USA). The sequences obtained from the positive samples were submitted to the BLASTn program for nucleotide analysis in comparison with sequences from the international database (GenBank). The quality of the sequences was analyzed using Geneious Prime (2020.2.1).

Although the amplified product contains 188 base pairs, only 75 base pairs could be used in the BLASTn analysis, showing 97.73% similarity with *Trypanosoma cruzi* (GenBank accession number: HM015660.1). However, the small fragment, along with the presence of two degenerate bases, makes a more accurate analysis impossible, but it confirms the amplification of the etiological agent as predicted by Moser et al. (1989).

In the present study, we reported *T. cruzi* infection in a six-banded armadillo, confirmed by PCR assay and genetic sequencing. A few studies have previously reported different infection rates of *E. sexcinctus* by *T. cruzi* using molecular assays (Orozco et al., 2013; Martins et al., 2015; Acosta et al., 2017; Santos et al., 2019a). However, the number of animals enrolled in these surveys was usually low, reflecting the difficulty in obtaining a substantial sample of free-ranging wild animals for epidemiological studies due to ethical issues. Thus, although the infection of armadillos with this protozoan has been sporadically reported, information regarding the specific role of this species in the epidemiological chain of Chagas disease remains unclear.

No evidence of infection with *Leishmania* spp., *Leptospira* spp., and *T. gondii* was found using PCR assays in the current study conducted on twenty armadillos from distinct locations. It is important to emphasize that a negative PCR result does not necessarily rule out infection, since some aspects such as the phase of infection, parasite/bacterial burden, and problems with PCR processing could influence the final result (Yang & Rothman, 2004). Since PCR for endogenous gene GAPDH was performed and DNA was detected in all samples, we believe that problems with PCR processing did occur in this study. A recent study reported seropositivity rates of 6%, 9% and 6% for *Leishmania* spp., *Leptospira* spp., and *T. gondii*, respectively, in a population of 33 free-ranging six-banded armadillos from Northeastern Brazil (Barbosa et al., 2020). Moreover, leptospires have been isolated from the urine and *T. gondii* has been identified in *E. sexcinctus* tissues (Vitaliano et al., 2014; Silva et al., 2015). These findings demonstrated the natural exposure of armadillos to these zoonotic agents, which are commonly observed affecting people and other animal species in the studied geographical area (Lacerda et al., 2008; Barbosa et al., 2009; Santos et al., 2019b; Rizzo et al., 2022; Murilo et al., 2023; Bezerra et al., 2024). However, further investigations are necessary to assess the importance of six-banded armadillos in the sylvatic cycle of these zoonoses, and to investigate their potential as a source of infection to humans, considering that the local population has a close relation with the hunting and meat consumption of these animals, mainly motivated by cultural reasons (Barboza et al., 2016).

Finally, it is important to highlight that this is the first study comprising a molecular investigation of different zoonotic pathogens in six-banded armadillos. Although different zoonotic agents have been sporadically identified in *E. sexcinctus*, we did not identify any molecular evidence of infection for most of the infectious agents assessed, except for *T. cruzi*, which was identified in only one animal. Until this point, the zoonotic infectious agent of more concern in six-banded armadillos is *M. leprae*, which was previously identified in all animals of a survey with 20 specimens conducted in Northeastern Brazil (Ferreira et al., 2020). It was expected to diagnose more zoonotic agents in armadillos, however, we realize that studies with this species are still scarce and the epidemiology of these diseases needs to be elucidated, what can be demonstrated through the different rates of infection reported in the studies (Orozco et al., 2013; Martins et al., 2015; Acosta et al., 2017; Santos et al., 2019a; Barbosa et al., 2020; Ferreira et al., 2020). Although these results could not be extrapolated to the overall six-banded armadillo population, the findings reported here bring new and important knowledge regarding zoonotic diseases in this species. Future studies, using a higher sample, during different climatic seasons, and for longer periods, are necessary to monitor and assess the epidemiology of these zoonotic pathogens in free-ranging six-banded armadillos.

## References

[B001] Acosta N, López E, Lewis MD, Llewellyn MS, Gómez A, Román F (2017). Hosts and vectors of *Trypanosoma cruzi* discrete typing units in the Chagas disease endemic region of the Paraguayan Chaco. Parasitology.

[B002] Alves FM, Lima JS, Rocha FL, Herrera HM, Mourão GM, Jansen AM (2016). Complexity and multi-factoriality of *Trypanosoma cruzi* sylvatic cycle in coatis, *Nasua nasua* (Procyonidae), and triatomine bugs in the Brazilian Pantanal. Parasit Vectors.

[B003] Barbosa IR, de Carvalho Xavier Holanda CM, de Andrade-Neto VF (2009). Toxoplasmosis screening and risk factors amongst pregnant females in Natal, northeastern Brazil. Trans R Soc Trop Med Hyg.

[B004] Barbosa WO, Coelho TG, Costa TO, Paiz LM, Fornazari F, Langoni H (2020). Antibodies against *Toxoplasma gondii, Leishmania* spp., and *Leptospira* spp. in Free-ranging Six-banded Armadillos (*Euphractus sexcinctus*) from Northeastern Brazil. J Wildl Dis.

[B005] Barboza RRD, Lopes SF, Souto WMS, Fernandes-Ferreira H, Alves RRN (2016). The role of game mammals as bushmeat in the Caatinga, northeast Brazil. Ecol Soc.

[B006] Bezerra JAB, Haisi A, Rocha GDS, Lima SG, Brasil AWL, Tomaz KLR (2024). Coinfection with *Leishmania infantu*m and *Toxoplasma gondii* in domestic cats from a region with a high prevalence of feline immunodeficiency virus. Microorganisms.

[B007] Birkenheuer AJ, Levy MG, Breitschwerdt EB (2003). Development and evaluation of a seminested PCR for detection and differentiation of *Babesia gibsoni* (Asian genotype) and *B. canis* DNA in canine blood samples. J Clin Microbiol.

[B008] Deps P, Antunes JM, Santos AR, Collin SM (2020). Prevalence of *Mycobacterium leprae* in armadillos in Brazil: A systematic review and meta-analysis. PLoS Negl Trop Dis.

[B009] Ferreira JS, de Carvalho FM, Pessolani MCV, Antunes JMAP, Oliveira IVPM, Moura GHF (2020). Serological and molecular detection of infection with *Mycobacterium leprae* in Brazilian six banded armadillos (*Euphractus sexcinctus*). Comp Immunol Microbiol Infect Dis.

[B010] Fornazari F, da Silva RC, Richini-Pereira VB, Beserra HE, Luvizotto MC, Langoni H (2012). Comparison of conventional PCR, quantitative PCR, bacteriological culture and the Warthin Starry technique to detect *Leptospira* spp. in kidney and liver samples of naturally infected sheep from Brazil. J Microbiol Methods.

[B011] Fornazari F, Langoni H, Marson PM, Nóbrega DB, Teixeira CR (2018). *Leptospira* reservoirs among wildlife in Brazil: beyond rodents. Acta Trop.

[B012] Homan WL, Vercammen M, De Braekeleer J, Verschueren H (2000). Identification of a 200- to 300-fold repetitive 529 bp DNA fragment in *Toxoplasma gondii*, and its use for diagnostic and quantitative PCR. Int J Parasitol.

[B013] Lacerda HG, Monteiro GR, Oliveira CC, Suassuna FB, Queiroz JW, Barbosa JD (2008). Leptospirosis in a subsistence farming community in Brazil. Trans R Soc Trop Med Hyg.

[B014] Martins K, Andrade CM, Barbosa-Silva AN, do Nascimento GB, Chiari E, Galvão LM (2015). *Trypanosoma cruzi* III causing the indeterminate form of Chagas disease in a semi-arid region of Brazil. Int J Infect Dis.

[B015] Mérien F, Amouriaux P, Perolat P, Baranton G, Saint Girons I (1992). Polymerase chain reaction for detection of *Leptospira* spp. in clinical samples. J Clin Microbiol.

[B016] Moser DR, Kirchhoff LV, Donelson JE (1989). Detection of *Trypanosoma cruzi* by DNA amplification using the polymerase chain reaction. J Clin Microbiol.

[B017] Murilo BMC, Andrade FP, Cordeiro LV, Barbosa VSA (2023). Epidemiological aspects of visceral leishmaniasis in Rio Grande do Norte, Brazil. J Trop Pathol.

[B018] Orozco MM, Enriquez GF, Alvarado-Otegui JA, Cardinal MV, Schijman AG, Kitron U (2013). New sylvatic hosts of *Trypanosoma cruzi* and their reservoir competence in the humid Chaco of Argentina: a longitudinal study. Am J Trop Med Hyg.

[B019] Rizzo H, Rocha LLL, Diniz DDM, Lima GS, Jesus TKS, Pinheiro JW (2022). Seroprevalence of *Leptospira* spp. in horses from Rio Grande do Norte, Brazil. Pesq Vet Bras.

[B020] Santos FM, Barreto WTG, de Macedo GC, Barros JHDS, Xavier SCDC, Garcia CM (2019). The reservoir system for *Trypanosoma* (Kinetoplastida, Trypanosomatidae) species in large neotropical wetland. Acta Trop.

[B021] Santos IMC, Leite AI, Furquim MEC, Zanatto DCS, Fernandes SJ, Silva GCP (2019). Frequency of antibodies and risk factors associated with *Toxoplasma gondii* infection in backyard pig production in the city of Mossoró, state of Rio Grande do Norte, Brazil. Rev Bras Parasitol Vet.

[B022] Silva FJ, Santos CEP, Silva TR, Silva GCP, Loffler SG, Brihuega B (2015). Search of leptospires and of antibodies against leptospires in animals and human beings in farms in Pantanal and Caatinga Brazilian biomes. Braz J Vet Res Anim Sci.

[B023] Vitaliano SN, Soares HS, Minervino AHH, Santos ALQ, Wether K, Marvulo MFV (2014). Genetic characterization of *Toxoplasma gondii* from Brazilian wildlife revealed abundant new genotypes. Int J Parasitol Parasites Wildl.

[B024] Volpini AC, Passos VM, Oliveira GC, Romanha AJ (2004). PCR-RFLP to identify *Leishmania* (*Viannia*) *braziliensis* and *L.* (*Leishmania*) *amazonensis* causing American cutaneous leishmaniasis. Acta Trop.

[B025] Yang S, Rothman RE (2004). PCR-based diagnostics for infectious diseases: uses, limitations, and future applications in acute-care settings. Lancet Infect Dis.

